# Bacteriophages in the gastrointestinal tract and their implications

**DOI:** 10.1186/s13099-017-0196-7

**Published:** 2017-08-10

**Authors:** Marzanna Łusiak-Szelachowska, Beata Weber-Dąbrowska, Ewa Jończyk-Matysiak, Renata Wojciechowska, Andrzej Górski

**Affiliations:** 1 Laboratory of Bacteriophages, Hirszfeld Institute of Immunology and Experimental Therapy, Polish Academy of Sciences (HIIET PAS), 53-114 Wrocław, Poland; 2 Department of Clinical Immunology, Transplantation Institute, Medical University of Warsaw, 02-006 Warsaw, Poland

**Keywords:** Bacteriophages, Gut microbiota, IBD, Phage translocation, Role of phages in the gut

## Abstract

The gut microbiota plays an essential role in health and disease of humans. Bacteriophages are the most abundant members of the gut virobiota and display great diversity. Phages can translocate through the mucosa to lymph and internal organs and play a role as regulators of the bacterial population in the gut. Increasing abundance of phages in the gut mucosa may reduce colonization by bacteria. Moreover, phages may have an immunomodulatory role in the immune response in the human gut. The role of phages in inflammatory bowel disease (IBD) remains unknown. Phages may take part in the development of IBD, but there are also data suggesting the protective role of phages in the gut of patients with IBD. Furthermore, recent data suggest that phages may mediate the beneficial effects of fecal microbiota transplantation (FMT). Therefore, evidence is accumulating to highlight the protective immunomodulating activity of the gut phages.

## Background

The gut microbiota consists of various microorganisms including bacteria (bacterial microbiota), bacteriophages and eukaryotic viruses (virobiota) and other microorganisms (fungi and archaea). Bacteriophages—bacterial viruses that attack specific bacteria—have been found in both animal and human organisms (oral, gastrointestinal and respiratory tracts, urine and serum) [1, 2]. Phages constitute an alternative to the use of antibiotics and are particularly applicable in the treatment of antibiotic-resistant bacterial infections [3–5]. Bacteriophages replicate by two alternative cycles: lytic and lysogenic life cycles [6]. In the lytic cycle phages infect bacteria leading to the production of new phage particles and degradation (lysis) of bacteria. A single phage particle adsorbs to a bacterial cell, then multiplies within. Progeny phages lyse the bacterial cell and release approximately 50–100 phage particles in one growth cycle [7, 8]. During the lysogenic cycle the genetic material of the phage may integrate into the bacterial genome, a process which does not lead to lysis of the bacteria [6]. In recent years there has been observed growing interest in the human gut microbiota and its effects on health and disease of humans [1, 9, 10]. It is suggested that gut microbiota may have an influence on the development of metabolic inflammation in obesity and diabetes [11]. Detection of phages may be performed by traditional bacterial lysis methods [12]. Bacteriophage abundance, diversity and morphology are investigated in transmission electron microscopy [13]. In recent years, researchers have begun to characterize the diversity of viruses using metagenomic methods [1, 10, 13]. A huge number of phages in the intestine have been investigated using electron microscopy, and their diversity has been investigated in metagenomic studies [13].

## Phages in the gut of healthy individuals

In the first months of an infant’s life, the intestinal bacteriophage virobiota is composed of a great number and variety of bacteriophages mainly from double-stranded DNA viruses belonging to the order *Caudovirales* [14]. The richness of phages decreases over the first 2 years of life, and single-stranded DNA viruses belonging to the family *Microviridae* come to dominate. Various factors such as geographical distribution and diet may affect the composition of phages in the gut. The intestinal microbiota plays an essential role in the development of healthy infants and affects health and disease [14]. Bacteriophages can influence the bacterial structure of the microbiome by attack and cause lysis of bacterial cells. Most of the gut phages in healthy adult people belong to the order *Caudovirales* with double-stranded DNA (*Podoviridae*, *Siphoviridae* and *Myoviridae*) or single-stranded DNA viruses from the families *Microviridae* and *Inoviridae* [9, 15–17]. A metagenomic study detected domination of the bacteria *Bacteroidetes* and *Firmicutes* in stool DNA from healthy human adults [9, 18]. Bacteriophages are most abundant in the virobiota fraction in the gut of healthy individuals, and they show large diversity [19]. Breitbart et al. [19] detected 1200 viral genotypes in the human stool from a single healthy adult. Most of them belonged to the family *Siphoviridae* (52.8%) and prophages (28.3%). Other studies have demonstrated that phages from the families *Siphoviridae*, *Myoviridae*, *Podoviridae* and prophages were most abundant in fecal virobiota from healthy individuals [15, 16, 20]. Muniesa and Jofre [13] estimated that the number of bacterial species in human feces is up to 800. The active bacteriophage virobiota in the gut of healthy humans consists of 35–2800 viruses [9], but metagenomic studies suggest that temperate phages dominate in the distal intestine [20]. It was demonstrated that the number of microorganisms in the gut increases from the proximal part to the distal. The concentration of virus-like particles (VLPs) in a human stool is estimated as 10^8^–10^9^ VLPs/g and ~10^9^ bacterial cells per gram of stool [15]. The number of viruses in the human microbiota does not always reflect the number of host bacteria [1]. The ratio of viruses to bacteria in the gut feces is low (0.1) in comparison to other environments, e.g. in the ocean (5–10) [15]. The gut bacteriophage virobiota was stable within a healthy individual over time [1, 9]. Ly et al. [21] investigated the diversity of fecal viruses between different households. Most of them were bacteriophages. Twenty healthy subjects were included in the study. From eight households consisting of two individuals one received treatment with an antibiotic (amoxicillin or azithromycin) for 7 days and the other received a placebo (vitamin C). Four controls did not receive any therapy. Each subject donated saliva and feces at day 0, day 3, day 7, week 8 and month 6. Use of relatively narrow spectrum oral antibiotics (amoxicillin or azithromycin) does not affect the composition of viruses in the gut. Similar patterns of viruses in the gut were determined between the members of each household, likely through transmissions from one individual to another. Fecal viruses were persistent over time in households [21].

## Phages in the gut of patients with intestinal diseases

Virulent phages were commonly found in the gut of patients with intestinal diseases. These phages may derive from induction of prophages from intestinal bacteria under stress conditions. Prophage induction may contribute to the occurrence of intestinal dysbiosis, changing the ratio of symbionts to pathobionts [22]. The use of some antibiotics can affect the induction of phages from lysogenic bacteria. Fluoroquinolones such as norfloxacin and ciprofloxacin may induce the production of phages from lysogenic bacterial strains due to the effect on DNA replication [23, 24]. Interestingly, Davies et al. [23] reported that fluoroquinolone treatment increases production of *Clostridium difficile* phages in human feces. Also *P. aeruginosa* phage was induced by antibiotics and oxidative stress in cystic fibrosis patient sputa. Other antibiotics such as colistin and meropenem inhibited the production of phages due to interruption of the cell membrane [24].

Factors that may cause the occurrence of inflammatory bowel diseases (IBD) are genetic factors, microbial factors and the immune response [25]. Lucas López et al. [26] reported the decrease in diversity and abundance of the bacteria *Bacteroidetes* and *Firmicutes* in feces/mucosa, microbiota associated with IBD. Moreover, bacteriophages may control the bacterial population in the human gut, influencing bacterial diversity and metabolism, but the role of phages in IBD remains to be examined. Metagenomic studies have shown that in Crohn’s disease dysbiosis of bacteria occurs [1, 27]. More VLPs were observed in the gut mucosa in Crohn’s disease compared to the healthy control (2.9 × 10^9^ vs 1.2 × 10^8^ VLPs/biopsy), and a decrease in viral diversity in Crohn’s disease was observed, which can affect the disease. Metagenomic analysis of bacteriophages in the gut of children with Crohn’s disease indicated diversity of phages, and the high abundance of phages suggested the participation of phages in the pathogenesis of Crohn’s disease [28]. Metagenomic analysis showed an increase in the richness of *Caudovirales* phages in the intestine in patients with IBD [29]. No increase in richness or diversity of *Microviridae* phages was detected in IBD. The authors investigated the differences between the virobiota in Crohn’s disease and ulcerative colitis. They observed an increase in richness of *Caudovirales* phages and higher phage diversity in Crohn’s disease, but such a correlation was not observed in the virobiota of patients with ulcerative colitis. The study supports the hypothesis that the virobiota may participate in intestinal inflammation and dysbiosis of bacteria. Lysis of bacteria leads to release of proteins, lipids and nucleic acids, which induce intestinal inflammation. Contradictory data were obtained by Manrique et al. [9], who reported that the number of bacteriophages decreases in inflammatory bowel disease, suggesting their role in the proper state of human health. Data concerning the presence of coliphages in stools of patients with IBD indicated lower frequencies of coliphages in comparison to volunteers [30]. The concentrations of coliphages were higher in stools of patients compared to healthy controls. The studies of coliphage presence in the gut of patients with IBD could suggest an association between lower phage presence and inflammatory bowel diseases.

## Phage translocation and potential implications of this phenomenon

It has been suggested by our group and then confirmed by others that viruses can translocate across the mucosal barriers and migrate to peripheral blood and local tissues and influence the immune system [1, 31]. In the blood of healthy individuals and immune suppressed patients there were detected phages belonging to the order *Caudovirales* [1]. Phage translocation can be higher in patients, because in many diseases affecting the gastrointestinal tract the gut barrier is more permeable to microorganisms [31]. Weber-Dąbrowska et al. [32] suggested phage presence in most of the tested blood samples after 10 days of oral phage therapy in patients with bacterial infections. However, Bruttin and Brüssow [33] did not detect phages in the plasma samples of healthy volunteers after one month of T4 phage oral administration. However, the phage was detected in the feces of all volunteers using a phage dose of 10^5^ PFU/ml. Their study suggests stability of the phage during gastrointestinal transit. What is more, the number of *E. coli* bacteria in feces did not decrease after oral phage administration. Recently Burcelin [11] described the mechanism of bacterial translocation from the gut to tissue, which induces inflammation and development of metabolic disease. Phages in the gut may have some immunoregulatory effect through the inhibition of the local immune responses to antigens from the gut flora [31]. Phages can influence bacterial translocation by destroying gut bacteria and can inhibit gut inflammation caused by bacterial translocation. Phages in the gut may downregulate gut immune cells such as dendritic cells and prevent proinflammatory action of these cells [5, 34].

Phages are not only known as regulators of the bacterial population in the gut but can also play a role in other parts/organs of the human or animal organism [35]. It is assumed that phages can move, like bacteria, between different parts of the body. They were detected in approximately 45% of clinical samples of ascitic fluid and urine from patients with potential microbial infections, while they were not found to be present in samples of blood, serum or cerebrospinal fluid. The researchers explained that the cerebrospinal fluid samples did not contain phages because translocation seldom occurs there. Phages are detected in blood or serum rarely or never, because of the low density of phages in these samples. Further confirmation of the translocation and immunomodulating hypothesis has recently been provided by Thannesberger et al. [36], who—using novel metagenomic approaches—showed the presence of phages in urine of healthy individuals as well as patients. Therefore, by translocating from the gut to other tissues phages may mediate their immunomodulating functions not only locally in the intestinal tract, but also at other sites of the mammalian organism.

## The potential role of intestinal phages

**Fig. 1:**
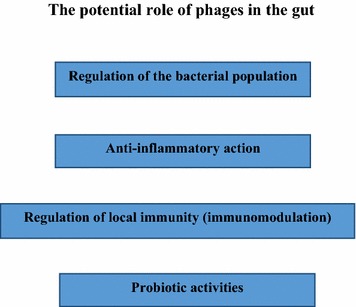
The potential importance of intestinal phages in the gut. Phages can regulate the bacterial population of the gut microbiota. Furthermore, they can also mediate anti-inflammatory action, not only by mere elimination of bacterial pathogens, but also by direct interactions with cells producing proinflammatory cytokines and reducing the overproduction of reactive oxygen species, thereby downregulating oxidative stress. Phage interactions with gut-associated lymphoid tissue may cause protective immunomodulating effects. Those and other phage effects may be similar to the health benefits provided by probiotics

It is believed that phages may play an important role in human immunity by defense of the mucosal barrier against bacteria [1]. Phages populate the mucosa to a greater degree than bacteria [37]. Phage-to-bacteria ratios were higher in mucosal surfaces than in the surrounding environment in samples from invertebrates and vertebrates. Studies of tissue culture cells in vitro showed that an increase of phage abundance in the mucosa protects the epithelium against bacteria. Phages interact with mucin glycoproteins in mucus via Ig-like domains exposed on the phage capsid [37]. Furthermore, mucus-adherent phages may influence both innate and acquired immunity [38]. Mucus-adherent phages may decrease colonization of the mucosa by the bacteria more than nonadherent phages [39]. Temperate phages present in the mucosa may protect the bacterial host against the same or related phages, but also this type of phages may participate in the selection of commensal microbiota [38]. Investigations carried out on experimental animals and patients undergoing phage therapy suggest that phages may reveal anti-bacterial and also anti-inflammatory action that can be advantageous clinically [5]. A schema of the role of intestinal phages in the gut is shown in Fig. 1. What is more, it was suggested and at least partly confirmed that phages may play an immunomodulatory role in the immune response in the gut against bacteria, viruses and cancer [5, 40]. Their immunomodulatory activity was observed also as direct action on T and B cells [34]. Phages may have an immunosuppressive role in the gut through participation in controlling inflammatory and autoimmune reactions [2]. This opens the way to potential application of phages in diseases where intestinal inflammation plays a significant role, therefore application of phage therapy in bowel diseases needs to be investigated. Phage therapy may potentially be used in treatment of IBD, where an adherent-invasive *E. coli* strain contributes to the disease in animal models [41]. Recently reported data suggest that in patients with recurrent *C. difficile* infection, bacterial microbiota and the virobiota can be pathologically altered [42]. It is well known that fecal microbiota transplantation (FMT) may bring about a healthy state and bring a significant clinical improvement. The patient’s phages in the gut indicated stability and donor-similar characteristics after FMT up to 7 months [42]. The recent publication of Ott et al. [43] describes greater efficacy of sterile fecal filtrate transfer than fecal microbiota transplantation in reduction of symptoms in patients with *C. difficile* infection. Sterile fecal filtrate recovered from normal stool contains a complex of bacteriophages, as shown by analysis of VLPs from the filtrate, which suggests that phages can mediate the beneficial effects of FMT.

## Conclusions

Accumulating data strongly suggest that intestinal phages can have an important role in protecting the host from pathogens. This also includes anti-inflammatory and immunomodulatory activity of phages. Further studies are necessary to throw more light on immunomodulatory phage functions and how they can be translated into new treatment modalities.

## Abbreviations

IBD: inflammatory bowel disease
VLPs: virus-like particles
PFU: plaque-forming units
FMT: fecal microbiota transplantation
